# Antithrombotic Treatments in Patients with Chronic Coronary Artery Disease or Peripheral Artery Disease: A Systematic Review of Randomised Controlled Trials

**DOI:** 10.1155/2020/3057168

**Published:** 2020-06-23

**Authors:** Rupert Bauersachs, Olivia Wu, Jean-Baptiste Briere, Kevin Bowrin, Katarzyna Borkowska, Anna Jakubowska, Vanessa Taieb, Mondher Toumi, Maria Huelsebeck

**Affiliations:** ^1^Klinikum Darmstadt GmbH, Darmstadt, Germany; ^2^Health Economics and Health Technology Assessment Research Group, University of Glasgow, Glasgow, UK; ^3^Bayer AG, Berlin, Germany; ^4^Bayer Plc, Reading, UK; ^5^Creativ-Ceutical, Krakow, Poland; ^6^Creativ-Ceutical, London, UK; ^7^Department of Public Health, Aix-Marseille University, Marseille, France

## Abstract

**Aims:**

Acetylsalicylic acid (ASA) is widely used for the prevention of atherothrombotic events in patients with chronic coronary artery disease (CAD) and peripheral artery disease (PAD), but the risk of vascular events remains high. We aimed at identifying randomised controlled trials (RCTs) on antithrombotic treatments in patients with chronic CAD or PAD.

**Methods:**

Searches were conducted on MEDLINE, EMBASE, and CENTRAL on March 1^st^, 2018. This systematic review (SR) uses a narrative synthesis to summarize the evidence for the efficacy and safety of antiplatelet and anticoagulant therapies in the population of both chronic CAD or PAD patients.

**Results:**

Four RCTs from 27 publications were included. Study groups included 15,603 to 27,395 patients. ASA alone was the most extensively studied (*n* = 3); other studies included rivaroxaban with or without ASA (*n* = 1), vorapaxar alone (*n* = 1), and clopidogrel with (*n* = 1) or without ASA (*n* = 1). Clopidogrel alone and clopidogrel plus ASA compared to ASA presented similar efficacy with comparable safety profile. Rivaroxaban plus ASA significantly reduced the risk of the composite of cardiovascular death, myocardial infarction, and stroke compared to ASA alone, although major bleeding with rivaroxaban plus ASA increased.

**Conclusion:**

There is limited and heterogeneous evidence on the prevention of atherothrombotic events in patients with chronic CAD or PAD. Clopidogrel alone and clopidogrel plus ASA did not demonstrate superiority over ASA alone. A combination of rivaroxaban plus ASA may offer significant additional benefit in reducing cardiovascular outcomes, yet it may increase the risk of bleeding, compared to ASA alone.

## 1. Introduction

Atherosclerosis is one of the most common causes of mortality and morbidity worldwide. It can affect any artery in the body including arteries in the heart, brain, arms, legs, pelvis, and kidneys. Atherosclerosis may cause CAD when there is a restriction of blood flow to the heart, or PAD, which causes stenosis and occlusion of noncerebral and noncoronary arteries affecting the arteries of the lower extremities and sometimes the carotid arteries [1, 2]. CAD or PAD poses a substantial clinical and economic burden, being associated with a high risk of cardiovascular (CV) death, MI, and stroke, which increases further in patients with prior MI, stroke, or renal impairment.

Acetylsalicylic acid (ASA) alone represents the standard of care for the prevention of atherothrombotic events in patients with chronic CAD or PAD. Despite the wide use of ASA, which is effective in the prevention of atherothrombotic events in patients with chronic CAD or PAD, the risk of vascular events remains high [3–6].

Rivaroxaban is an oral factor Xa inhibitor anticoagulant which has been approved for various indications [7–14]. Recently, the COMPASS study conducted with the aim to investigate the efficacy of rivaroxaban in preventing recurrent cardiovascular events in the population of both chronic CAD or PAD patients showed that rivaroxaban 2.5 mg twice a day (bid) plus ASA effectively prevents atherothrombotic events [15]. Rivaroxaban 2.5 mg, coadministered with ASA, is indicated for the prevention of atherothrombotic events in adult patients with coronary artery disease (CAD) or symptomatic peripheral artery disease (PAD) at high risk of ischaemic events [16].

## 2. Aim

The aim of this systematic review (SR) was to identify and summarise the current evidence on therapeutic approaches in the prevention of atherothrombotic events in the population of both chronic CAD or PAD patients focusing on randomised controlled trials (RCTs).

## 3. Methods

We conducted an SR of RCTs assessing the efficacy and safety of the anticoagulants and antiplatelet therapies in the prevention of atherothrombotic events in the population of both chronic CAD or PAD patients. The PRISMA guidelines were followed [17]. The protocol of the SR was not preregistered in PROSPERO.

Detailed inclusion and exclusion criteria are provided in Table 1. To ensure the similar quality of evidence found and because of the specifics of the population, observational studies were excluded. The searches were conducted via the OVID interface on March 1, 2018, in MEDLINE® (1946 to present) and EMBASE® (1974 to present) and via Wiley Library in the Cochrane Central Register of Controlled Trials (CENTRAL). Reference lists of relevant studies were manually searched for additional articles. No restrictions in terms of language, timeframe, or geographical scope were applied. Conference abstracts published before 2015 were excluded, as the results of these studies are likely to have been already released as manuscripts.

The search and selection of studies were performed by two reviewers; when necessary, a third reviewer resolved any discrepancies (VT, KBO, and AJ). The search strategies are detailed in Tables S1 and S2. Extracted data included publication characteristics, study details, patient characteristics, results, and study limitations. A reviewer extracted data from selected publications and the quality of the extraction was evaluated by a second reviewer (VT, KBO, and AJ). The clinicaltrial.gov was searched to supplement the data found in the publications. The risk of bias of the included studies was assessed using the RoB 2.0 tool [18].

Due to the high level of heterogeneity of the included population and interventions, statistical meta-analysis was not conducted.

## 4. Results

**Figure 1:**
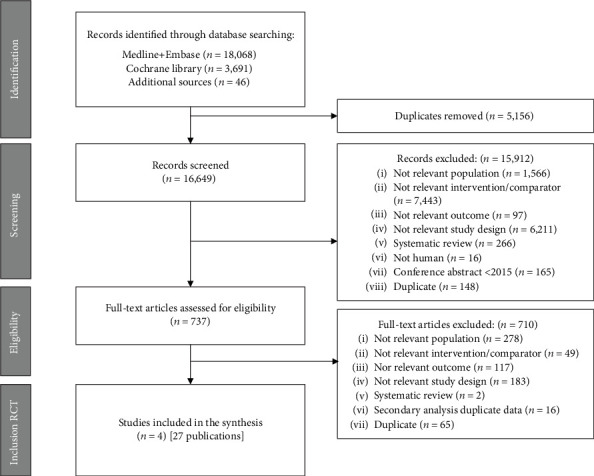
PRISMA flow chart.

A total of 21,805 articles were identified: 21,759 from the electronic databases including MEDLINE, EMBASE, and CENTRAL and 46 from additional sources that were publications obtained through reference screening of relevant studies (Figure 1). A total of 27 publications reporting the results of 4 studies (Table 2) were included in the narrative synthesis.

### 4.1. Study and Patient Characteristics

Detailed study characteristics are provided in Table 2. All of the included studies were international and sponsored by pharmaceutical companies. The trial phase was reported for 3 studies; all 3 studies were reported as phase III trials. Studies randomised from 15,603 to 27,395 patients. The largest study identified was the COMPASS study [15]. The proportion of men was between 70% and 80%. Three studies reported the mean age of the population examined. Of these, 1 study included patients aged below 65 years on average [19], while 2 other studies included patients between 65 and 74 years [1, 15].

The mean follow-up in all studies exceeded 12 months. Vorapaxar vs. placebo [20] was investigated at the most extended follow-up (the median of 30 months of follow-up), followed by the study of clopidogrel plus ASA vs. ASA alone [1], rivaroxaban with and without ASA vs. ASA alone [15], and clopidogrel alone vs. ASA alone [19].

As it is the current standard of care, ASA alone was the most examined treatment across the identified studies (*n* = 3). Rivaroxaban (alone and plus ASA), vorapaxar alone, and clopidogrel (alone and plus ASA) were investigated in single studies. The highest dose of ASA (325 mg once daily (od)) was examined in the CAPRIE study [19]. The COMPASS [15] and CHARISMA [1] studies investigated doses of ASA at 100 mg od and 75-162 mg od, respectively.

Inclusion criteria in identified studies varied significantly. The COMPASS [15] study included patients with chronic atherosclerotic vascular disease, while patients with recent nonlacunar ischaemic stroke were excluded. The CHARISMA [1] study randomised a broader population, including chronic patients with either documented CAD, symptomatic PAD, cerebrovascular disease, or multiple atherothrombotic risk factors. The CAPRIE [19] trial recruited a population including both chronic and acute patients, consisting of subjects who were diagnosed with PAD or experienced an ischaemic stroke from at least 1 week up to 6 months before enrolment or MI within up to 35 days before enrolment. Detailed study inclusion criteria are shown in Table S3.

Although all the studies enrolled patients with chronic CAD or PAD, only 3 of them reported the proportion of patients with either disease. The percentage of patients with chronic CAD ranged from 47.7% [1] to 90.8% [15]. The proportion of patients with PAD ranged from 21.9% [20] to 27.4% [15].

All of the included studies reported data on the percentage of patients with stroke/cerebrovascular disease, which ranged from 3.7% [15] to 35.6% [1].

Outcome definitions in included studies varied. It was particularly evident for major bleeding. In the COMPASS trial [15], the definition of major bleeding was based on the modified International Society on Thrombosis and Haemostasis (ISTH) criteria, while the remaining studies defined the event according to the ISTH [20], Thrombolysis in Myocardial Infarction (TIMI) classification [20], the Global Utilization of Streptokinase and Tpa for Occluded Arteries (GUSTO) [1] criteria, or other adopted trial-specific criteria [19].

All included studies were double-blinded trials, and all of them reported detailed information on sequence generation method and allocation concealment. Intention to treat population was a primary analysis set in all eligible studies—risk of bias due to missing outcome data was low. There was no evidence to suggest the selective outcomes reporting in all included studies. The assessment of risk of bias of all included studies is presented in Table S4.

### 4.2. Study Results

**Figure 2:**
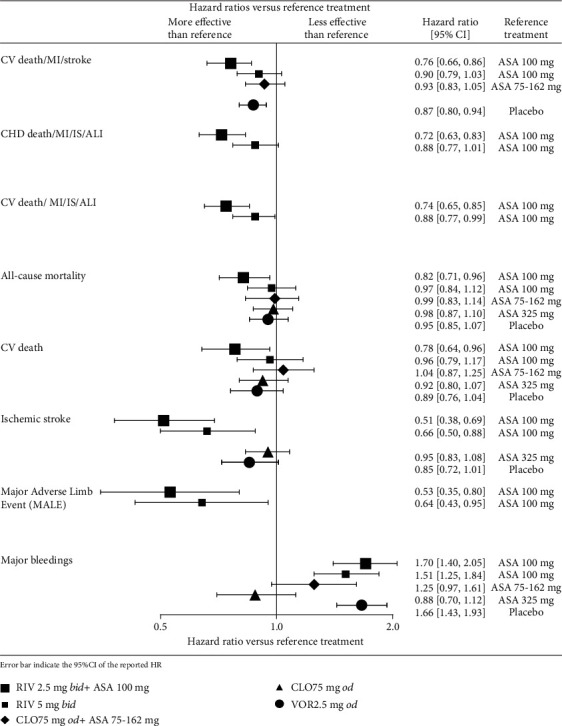
Forest plot with key efficacy and safety results [1, 15, 19, 20]. ASA: acetylsalicylic acid; CLO: clopidogrel; RIV: rivaroxaban; trt: treatment; VOR: vorapaxar.

The present SR focused on the outcomes of clinical relevance—composites of major adverse cardiovascular events, all-cause mortality, CV death, ischaemic stroke, major adverse limb events (MALE), and major bleedings in the light of the antiplatelet and anticoagulant medication strategies. The results are presented in Table 3 and on Figure 2. Additional results are presented in Table S5. The full list of included studies with corresponding publications is presented in Table S6.

#### 4.2.1. Clopidogrel Alone

The efficacy and safety of clopidogrel alone compared to ASA alone were reported in the CAPRIE study [19]. At a median time of follow-up of 22.9 months, no significant difference in ischaemic stroke, all-cause mortality, and CV death was observed between clopidogrel alone and ASA alone. The risk of major bleeding was comparable between clopidogrel alone and ASA alone.

#### 4.2.2. Clopidogrel Plus ASA

The efficacy and safety of clopidogrel plus ASA compared to ASA alone were investigated in the CHARISMA [1] study. At a median follow-up of 28 months, no difference between clopidogrel plus ASA and ASA alone was identified in the composite of CV death, MI, and stroke, as well as all-cause mortality, and CV death. The risk of major bleeding was comparable between clopidogrel plus ASA and ASA alone.

#### 4.2.3. Rivaroxaban Plus ASA

The efficacy and safety of rivaroxaban plus ASA compared to ASA alone was investigated in the COMPASS study [15], with a mean follow-up of 23 months. The risk of the composite of CV death, MI, and stroke was significantly lower for rivaroxaban plus ASA in comparison to ASA alone. The secondary composite outcomes (coronary heart disease death, MI, ischaemic stroke, acute limb ischaemia, and CV death, MI, ischaemic stroke, acute limb ischaemia) occurred in fewer patients in the rivaroxaban plus ASA group compared to the ASA alone group. Rivaroxaban plus ASA was associated with a nonsignificant difference in all-cause mortality. Rivaroxaban plus ASA compared to ASA alone significantly reduced the risk of CV death and ischaemic stroke. Compared to ASA alone, rivaroxaban plus ASA significantly lowered the incidence of MALEs. Major bleeding with rivaroxaban 2.5 mg bid plus ASA was significantly increased compared to ASA alone.

#### 4.2.4. Rivaroxaban Alone

The efficacy and safety of rivaroxaban alone compared to ASA alone were investigated in the COMPASS study [15], with a mean time of follow-up of 23 months. No significant difference was identified in terms of the incidence of the composite of CV death, MI, and stroke, and the composite of coronary heart disease death, MI, and ischaemic stroke, and the risk of all-cause mortality for rivaroxaban alone compared to ASA alone. Rivaroxaban alone significantly reduced the incidence of the composite of CV death, MI, and ischaemic stroke, and acute limb ischaemia, the incidence of MALEs in comparison to ASA alone as well as the incidence of ischaemic stroke. The rate of major bleedings was significantly higher in the rivaroxaban group than in the ASA group.

#### 4.2.5. Vorapaxar

The efficacy and safety of vorapaxar was compared to placebo in the TRA 2°P–TIMI 50 [20] study with a median follow up of 30 months. Compared to placebo, vorapaxar significantly reduced the incidence of composite MI, stroke, and CV death, with no significant reduction in ischaemic stroke, all-cause mortality, as well as CV death. Vorapaxar showed a higher incidence of major bleeding when compared to placebo.

## 5. Discussion

Recently, several SRs were published that focused on pure CAD [21–24] or pure PAD [25, 26] populations.

In an SR investigating different antiplatelet agents for the prevention of major cardiovascular events and leg amputations in patients with PAD showed that clopidogrel should be the indicated antiplatelet agent in this group of patients [26]. Another study showed that dual antiplatelet therapy (DAPT) is beneficial for preventing thrombosis after revascularisation in PAD comparing to ASA monotherapy [25]. DAPT is also recommended after drug-eluting stent implantation; however, the optimum duration remains uncertain [23]. In a group of patients treated beyond 1 year, myocardial infarction and stent thrombosis were reduced; however, it was associated with increased mortality [23]. Another study showed that treatment of patients with acute coronary disease or prior myocardial infarction with DAPT (ASA plus ticagrelor) or triple therapy (ASA, clopidogrel, and very low dose rivaroxaban) was associated with lower cardiovascular mortality, but with increasing risk of major bleeding [22]. In an SR comparing ASA treatment with clopidogrel monotherapy in the treatment of stable CAD patients, no difference was found [24]. Furthermore the administration of prasugrel and ticagrelor led to a significant reduction in the incidence of major ischaemic events in the patients with CAD comparing to clopidogrel [21].

Although none of these SRs evaluated a broad effect on the population consist of both CAD and PAD patients. Taking this into consideration, this SR summarises the efficacy and safety of antiplatelet and anticoagulant regimens for the prevention of atherothrombotic events only in the population of both chronic CAD or PAD patients.

It was found that clopidogrel alone and clopidogrel with ASA compared to ASA alone presented similar efficacy in the prevention of atherothrombotic events with comparable safety profile. Vorapaxar treatment was associated with a lower incidence of composite MI, stroke, and CV death, with no significant reduction in mortality, compared to placebo. The rate of major bleeding was significantly increased in the vorapaxar group as compared with the placebo group. Rivaroxaban 5 mg bid did not show better cardiovascular outcomes than ASA alone and resulted in more major bleeding events. Rivaroxaban 2.5 mg bid plus ASA showed better efficacy when given in combination with ASA than ASA alone; however, this combined therapy increased the risk of major bleeding.

The current guidelines recommend ASA alone as the standard of care for the prevention of atherothrombotic events in patients with chronic CAD or PAD. In the chronic phase of the disease, patients should maintain single antiplatelet therapy with ASA (or clopidogrel in ASA-intolerant patients) to minimise the risk of atherothrombotic events as well as the risk of bleeding [3–6, 27, 28]. Among identified studies on the prevention of atherothrombotic events, the COMPASS [15] study on rivaroxaban was the largest (*n* = 27,395) study identified. The study was prematurely terminated due to the superiority of the rivaroxaban plus ASA group after a mean follow-up of 23 months.

As shown in the COMPASS study [15], patients with chronic CAD or PAD given a combination of rivaroxaban and ASA had significantly better prognosis when compared to patients receiving ASA alone. The rate of the primary outcome (a composite of CV death, MI and stroke) was lower by 24% with rivaroxaban plus ASA than with ASA alone (4.1% vs. 5.4%; *P* < 0.001), but the rate of major bleeding was higher (3.1% vs. 1.9%; *P* < 0.001). Earlier, two regimens of clopidogrel for secondary CV prevention in chronic CAD or PAD had been investigated: clopidogrel alone [19] and clopidogrel plus ASA [1].

In 1996, the CAPRIE study [19] including nearly 20,000 patients with either ischaemic stroke, MI, or symptomatic PAD did not demonstrate superiority of clopidogrel alone over ASA regarding either all-cause mortality or vascular-related mortality.

The third-largest study, CHARISMA [1], included nearly 16,000 patients with either documented CAD or symptomatic PAD, failed to demonstrate the superiority of dual antiplatelet therapy (DAPT) strategy based on clopidogrel plus ASA over ASA alone in terms of the composite of CV death, MI and stroke, all-cause mortality, and CV death [1]. The rate of severe bleeding did not differ significantly between these groups, although a strong trend towards an increased risk was observed in patients receiving clopidogrel plus ASA.

Rivaroxaban plus ASA compared to ASA alone significantly reduced the risk of ischaemic stroke (0.9% vs. 1.4%; *P* = 0.0035). Similar results were observed for the regiment of rivaroxaban alone versus ASA alone (0.7% vs. 1.4%, *P* = 0.00001) [15]. These results may suggest that for a population such as the COMPASS population including around 60% of prior MI patients, the prevention of ischaemic stroke in this population may be relevant.

Evidence identified for chronic CAD or PAD population is limited. We identified only 4 eligible studies; however, more than 15,000 hits were screened. No RCT on DAPT with ticagrelor was identified for the population of interest. Recently published studies that evaluated ticagrelor, EUCLID [29] and PEGASUS-TIMI 54 [30], were not included because they included only patients with symptomatic PAD and with prior myocardial infarction, respectively.

Only one study has an unclear risk of bias for the randomisation method and allocation concealment. Trials with unclear or inadequate concealment have been shown to report more favourable effects of experimental treatment [31].

Efficacy and safety of antiplatelets and anticoagulants in chronic CAD or PAD was investigated in heterogeneous patient population. The COMPASS [15] study included patients with chronic atherosclerotic vascular disease, while the CAPRIE [19] trial recruited a population including both chronic and acute patients, consisting of subjects who were diagnosed with PAD or experienced ischaemic stroke from at least 1 week up to 6 months before enrolment or MI within up to 35 days before enrolment.

The COMPASS study [15] used the definition of major bleeding according to modified ISTH criteria that were the broadest and also comprised of cases presented to an acute care facility without an overnight stay. Thus, comparison across studies should be interpreted with caution.

Follow-up duration in included studies varied from 22.9 [32] to 30 months [20]. The outcomes of short-term studies usually have several limitations resulting from inadequate power to demonstrate clinically relevant endpoints, lack of prespecified statistical hypotheses, and potential sensitivity to changes of hazards during follow-up.

## 6. Conclusion

This systematic review of efficacy and safety of interventions to prevent atherothrombotic events in patients with chronic CAD or PAD highlighted the limited evidence available and revealed population heterogeneity between trials. Clopidogrel alone and clopidogrel plus ASA did not demonstrate superiority over ASA alone. The addition of rivaroxaban to ASA may increase the risk of bleeding. Rivaroxaban and ASA may offer patients significant additional benefits compared to ASA alone.

## Figures and Tables

**Table 1:** Inclusion and exclusion criteria.

Domain	Inclusion criteria	Exclusion criteria
Population	Population of both chronic CAD or PAD patients	Children and adolescents (<18 years old) Studies evaluated only patients with CAD Studies evaluated only patients with PAD

Interventions	(i) Xaban/direct thrombin inhibitors (rivaroxaban, dabigatran, apixaban, edoxaban, betrixaban) (ii) Antiplatelet (ASA, clopidogrel, ticagrelor, ticlopidine, triflusal, and prasugrel) (iii) Dipyridamole (iv) Cilostazol (v) Vorapaxar (vi) Parenteral anticoagulants (low molecular weight heparin, unfractionated heparin, fondaparinux) (vii) Vitamin K antagonists (not limited to warfarin)	

Comparators	Interventions + placebo	

Outcomes of interest	Efficacy including: (i) Composite outcomes: (a) Stroke/MI/CV death (b) CHD death/MI/IS/ALI (c) CV death/MI/IS/ALI (ii) Individual components of composite outcomes (iii) All-cause mortality (iv) Amputations (v) HF (vi) Unstable angina (vii) Revascularisation (viii) Bypass graft failure (occlusion) (ix) Stent thrombosis (x) MALE (xi) Venous thromboembolism (xii) Haemorrhagic stroke (xiii) Chronic limb ischaemia	
	Safety including: (i) Major or severe bleeding (ii) Fatal bleeding (iii) Intracranial bleeding (iv) Bleeding requiring re-operation (v) Bleeding requiring hospitalisation (vi) Total serious AEs (vii) Total severe AEs (viii) Gastrointestinal bleeding (ix) Discontinuations (any, due to AEs)	

Study design	Study type: (i) RCTs (ii) Extensions of RCTs	Study type: (i) Review (ii) Opinion, editorial, letter

Publication type	Full-text papers, conference abstracts > 2014	Conference abstracts ≤ 2014

AEs: adverse events; ALI: acute limb ischaemia; CAD: coronary artery disease; CV: cardiovascular; HF: heart failure; IS: ischaemic stroke; MALE: major adverse limb events; MI: myocardial infarction; PAD: peripheral artery disease; RCT: randomised controlled trial; ASA: acetylsalicylic acid.

**Table 2:** Overview of included studies and patient characteristics (*N* = 4).

No.	Study	Study design	Sponsor	Country	Sample size	Follow-up (months)	Interventions	Male *n* (%)	Mean age (SD)	CAD *n* (%)	PAD *n* (%)	History of stroke/CV disease *n* (%)
1	CAPRIE [19]	Multicentre, double blind	Sanofi and Bristol-MyersSquibb	International (Australia, Austria, Belgium, Canada, Finland, France, Germany, Italy, Netherlands, New Zealand, Portugal, Spain, Sweden, Switzerland, UK, USA)	19,185	Mean: 22.9	Clopidogrel 75 mg od	(72)	62.5 (11.1)	–	–	[9]
							ASA 325 mg od	(72)	62.5 (11.1)	–	–	[9]

2	CHARISMA, NCT00050817 [1]	Multicentre, double blind, phase III study	Sanofi-Aventis and Bristol-Myers Squibb	International (Argentina, Australia, Austria, Belgium, Brazil, Canada, Chile, Czech Republic, Denmark, Finland, France, Germany, Greece, Hong Kong, Hungary, Italy, Malaysia, Mexico, Netherlands, Norway, Poland, Portugal, Russian Federation, Singapore, South Africa, Spain, Sweden, Switzerland, Taiwan, Turkey, UK, USA)	15,603	Median: 28	Clopidogrel 75 mg od+ASA 75-162 mg od	5,486 (70.3)	–	2,892 (47.70)	1,760 (22.60)	2,157 (35.60)
							ASA 75-162 mg od	5,473 (70.2)	–	2,943 (48.30)	1,771 (22.70)	2,163 (35.50)

3	COMPASS, NCT01776424 [15]	Multicentre, double blind, phase III study	Bayer	International (Argentina, Australia, Belgium, Brazil, Canada, Chile, China, Colombia, Czech Republic, Denmark, Ecuador, Finland, France, Germany, Hungary, Ireland, Israel, Italy, Japan, Malaysia, Netherlands, Philippines, Poland, Romania, Russia, Slovakia, South Africa, South Korea, Sweden, Switzerland, Ukraine, UK, USA)	27,395	Mean: 23	Rivaroxaban 5 mg bid	7,145 (78.4)	68.2 (7.9)	8,250 (90.50)	2,474 (27.10)	346 (3.80)
							Rivaroxaban 2.5 mg bid+ASA 100 mg od	7,093 (77.5)	68.3 (7.9)	8,313 (90.80)	2,492 (27.20)	351 (3.80)
							ASA 100 mg od	7,137 (78.2)	68.2 (8.0)	8,261 (90.50)	2,504 (27.40)	335 (3.70)

4	TRA 2°P–TIMI 50, NCT00526474 [20]	Multicentre, double blind, phase III study	Merck	International (Argentina, Australia, Austria, Belgium, Brazil, Canada, Chile, Colombia, Czech Republic, Denmark, Finland, France, Germany, Hong Kong, Hungary, Israel, Italy, Japan, Malaysia, Netherlands, New Zealand, Norway, Poland, Portugal, Puerto Rico, Singapore, South Africa, Spain, Sweden, Switzerland, UK, USA)	26,449	Median: 30	Vorapaxar 2.5 mg od	10,071 (76.2)	–	8,898 (67.3)	2,901 (21.9)	3,139 (23.70)
							Placebo	10,052 (76.0)	–	8,881 (67.2)	2,944 (22.3)	3,129 (23.70)

ASA: acetylsalicylic acid; bid: bis in die = twice a day; CAD: coronary artery disease; CV: cardiovascular; od: once a day; PAD: peripheral artery disease.

**Table 3:** Key efficacy and safety results.

Outcome	Study	Follow-up (months)	Interventions	*N*	*N* with event	% with event	Comparison HR (LCI; UCI)	*N*/100 p-yrs
CV death/MI/stroke	CHARISMA, NCT00050817 [1]	Median: 28	Clopidogrel 75 mg od+ASA 75-162 mg od	7802	534	6.80	0.93 (0.83; 1.05)	2.93^‡^
			ASA 75-162 mg od	7801	573	7.30	Ref.	3.15‡
	COMPASS, NCT01776424 [15]	Mean: 23	Rivaroxaban 5 mg bid	9117	448	4.90	0.90 (0.79; 1.03)	2.60^^^
			Rivaroxaban 2.5 mg bid+ASA 100 mg od	9152	379	4.10	0.76 (0.66; 0.86)	2.18^^^
			ASA 100 mg od	9126	496	5.40	Ref.	2.88^^^
	TRA 2°P–TIMI 50, NCT00526474 [20]	Median: 30	Vorapaxar 2.5 mg od	13225	1028	9.30^†^	0.87 (0.80; 0.94)	3.11^‡^
			Placebo	13224	1176	10.50†	Ref.	3.56^‡^

CHD death/MI/IS/ALI	COMPASS, NCT01776424 [15]	Mean: 23	Rivaroxaban 5 mg bid	9117	397	4.40	0.88 (0.77; 1.01)	2.31^^^
			Rivaroxaban 2.5 mg bid+ASA 100 mg od	9152	329	3.60	0.72 (0.63; 0.83)	1.89^^^
			ASA 100 mg od	9126	450	4.90	Ref.	2.62^^^

CV death/MI/IS/ALI	COMPASS, NCT01776424 [15]	Mean: 23	Rivaroxaban 5 mg bid	9117	453	5.00	0.88 (0.77; 0.99)	2.63^^^
			Rivaroxaban 2.5 mg bid+ASA 100 mg od	9152	389	4.30	0.74 (0.65; 0.85)	2.24^^^
			ASA 100 mg od	9126	516	5.70	Ref.	3.00^^^

All-cause mortality	CAPRIE [19]	Mean: 22.9	Clopidogrel 75 mg od	9599	560	3.00^†^	0.98 (0.87; 1.10)‡	3.18^‡^
			ASA 325 mg od	9586	571	3.10^†^	Ref.	3.26^‡^
	CHARISMA, NCT00050817 [1]	Median: 28	Clopidogrel 75 mg od+ASA 75-162 mg od	7802	371	4.80	0.99 (0.83; 1.14)	2.04^‡^
			ASA 75-162 mg od	7801	374	4.80	Ref.	2.05^‡^
	COMPASS, NCT01776424 [15]	Mean: 23	Rivaroxaban 5 mg bid	9117	366	4.00	0.97 (0.84; 1.12)	2.09^^^
			Rivaroxaban 2.5 mg bid+ASA 100 mg od	9152	313	3.40	0.82 (0.71; 0.96)	1.78^^^
			ASA 100 mg od	9126	378	4.10	Ref.	2.16^^^
	TRA 2°P–TIMI 50, NCT00526474 [20]	Median: 30	Vorapaxar 2.5 mg od	13225	540	5.00^†^	0.95 (0.85; 1.07)	1.63^‡^
			Placebo	13224	565	5.30^†^	Ref.	1.71^‡^

CV death	CAPRIE [19]	Mean: 22.9	Clopidogrel 75 mg od	9599	350	1.90^†^	0.92 (0.80; 1.07)^‡^	1.98^‡^
			ASA 325 mg od	9586	378	2.06^†^	Ref.	2.16‡
	CHARISMA, NCT00050817 [1]	Median: 28	Clopidogrel 75 mg od+ASA 75-162 mg od	7802	238	3.10	1.04 (0.87; 1.25)	1.31^‡^
			ASA 75-162 mg od	7801	229	2.90	Ref.	1.26^‡^
	COMPASS, NCT0177642 [15]	Mean: 23	Rivaroxaban 5 mg bid	9117	195	2.10	0.96 (0.79; 1.17)	1.11^^^
			Rivaroxaban 2.5 mg bid+ASA 100 mg od	9152	160	1.70	0.78 (0.64; 0.96)	0.91^^^
			ASA 100 mg od	9126	203	2.20	Ref.	1.16^^^
	TRA 2°P–TIMI 50, NCT00526474 [20]	Median: 30	Vorapaxar 2.5 mg od	13225	285	2.70^†^	0.89 (0.76; 1.04)	0.86^‡^
			Placebo	13224	319	3.00^†^	Ref.	0.96^‡^

IS	CAPRIE [19]	Mean: 22.9	Clopidogrel 75 mg od	9553	315^‡^	3.30^‡^	0.95 (0.83; 1.08)^‡^	1.79^‡^
			ASA 325 mg od	9546	338^‡^	3.50^‡^	Ref.	1.93^‡^
	COMPASS, NCT01776424 [15]^	Mean: 23	Rivaroxaban 5 mg BID	9117	83	0.90	0.66 (0.50; 0.88)	0.48^^^
			Rivaroxaban 2.5 mg BID+ASA 100 mg od	9152	64	0.70	0.51 (0.38; 0.69)	0.36^^^
			ASA 100 mg od	9126	125	1.40	Ref.	0.72^^^
	TRA 2°P–TIMI 50, NCT00526474 [20]	Median: 30	Vorapaxar 2.5 mg od	13225	250	2.20^†^	0.85 (0.72; 1.01)	1.23^‡^
			Placebo	13224	294	2.60^†^	Ref.	1.79^‡^

Major Adverse Limb Event (MALE)	COMPASS, NCT01776424 [15]	Mean: 23	Rivaroxaban 5 mg bid	9117	41	0.40	0.64 (0.43; 0.95)	0.23^^^
			Rivaroxaban 2.5 mg bid+ASA 100 mg od	9152	34	0.40	0.53 (0.35; 0.80)	0.19^^^
			ASA 100 mg od	9126	64	0.70	Ref.	0.37^^^

Major bleedings	CAPRIE^∗^ [19]	Mean: 22.9	Clopidogrel 75 mg od	9599	132	1.38	0.88 (0.70;1.12)^‡^	0.75^‡^
			ASA 325 mg od	9586	149	1.55	Ref.	0.85^‡^
	CHARISMA, NCT00050817^∗∗^ [1]	Median: 28	Clopidogrel 75 mg od+ASA 75-162 mg od	7802	130	1.70	1.25 (0.97; 1.61)	0.71^‡^
			ASA 75-162 mg od	7801	104	1.30	Ref.	0.57^‡^
	COMPASS, NCT01776424^∗∗∗^ [15]	Mean: 23	Rivaroxaban 5 mg bid	9117	255	2.8	1.51 (1.25; 1.84)	1.48^^^
			Rivaroxaban 2.5 mg bid+ASA 100 mg od	9152	288	3.1	1.70 (1.40; 2.05)	1.67^^^
			ASA 100 mg od	9126	170	1.9	Ref.	0.98^^^
	TRA 2°P–TIMI 50, NCT00526474^∗∗^ [20]	Median: 30	Vorapaxar 2.5 mg od	13186	438	4.20^†^	1.66 (1.43; 1.93)	1.33^‡^
			Placebo	13166	267	2.50^†^	Ref.	0.81^‡^
	TRA 2°P–TIMI 50, NCT00526474^∗∗∗∗^ [20]		Vorapaxar 2.5 mg od	13186	298	2.90^†^	1.44 (1.21; 1.72)	0.90^‡^
			Placebo	13166	209	1.90^†^	Ref.	0.63^‡^
	TRA 2°P–TIMI 50, NCT00526474^∗∗∗∗∗^ [20]		Vorapaxar 2.5 mg od	13186	624	5.90^†^	1.57 (1.38; 1.78)	1.89^‡^
			Placebo	13166	404	3.70^†^	Ref.	1.23^‡^

^∗^Trial-specific criteria definition, ^∗∗^GUSTO severe definition, ^∗∗∗^Modified ISTH definition, ^∗∗∗∗^TIMI major bleeding, ^∗∗∗∗∗^ISTH definition, ^‡^Calculated on the basis of available data, ^^^Unpublished data extracted from CSR, ^†^Kaplan-Meier estimate. ALI: acute limb ischaemia; ASA: acetylsalicylic acid; bid: bis in die = twice a day; CHD: coronary heart disease; CV: cardiovascular; GUSTO: Global Utilization of Streptokinase and Tpa for Occluded Arteries definition; HR: hazard ratio; IS: ischaemic stroke; ISTH: International Society on Thrombosis and Haemostasis classification; LCI: lower confidence interval; MI: myocardial infarction; od: once a day; p-yrs: Patients-years; Ref.: reference group; UCI: upper confidence interval.

## Supplementary Materials

Supplementary Materials Table S1: search strategy—RCT in Medline + Embase (via Ovid) (March 2018). Table S2: search strategy—CENTRAL (March 2018). Table S3: study inclusion criteria (*N* = 6). Table S4: risk of bias assessment (*N* = 6). Table S5: other efficacy and safety results. Table S6: list of included publications (*N* = 28, 6 studies).
