# Supplementary material for: Antithrombotic Treatments in Patients with Chronic Coronary Artery Disease or Peripheral Artery Disease: A Systematic Review of Randomised Controlled Trials
Source: Cardiovasc Ther. 2020 Jun 23;2020:3057168. doi: 10.1155/2020/3057168 (PMC7345917; doi:10.1155/2020/3057168)
Supplement: Supplementary Materials — Table S1: search strategy—RCT in Medline + Embase (via Ovid) (March 2018). Table S2: search strategy—CENTRAL (March 2018). Table S3: study inclusion criteria (N = 6). Table S4: risk of bias assessment (N = 6). Table S5: other efficacy and safety results. Table S6: list of included publications (N = 28, 6 studies). [file 3057168.f1.docx]

**Supplementary materials**

**Table S1 Search strategy – RCT in Medline + Embase (via Ovid) (March 2018)**

| ID | Search terms | Hits |
| --- | --- | --- |
| 1 | Coronary Artery Disease/ or Coronary Artery Disease.mp. or Peripheral Artery Disease/ or Peripheral Artery Disease?.mp. or Peripheral arterial Disease?.mp. | 376,714 |
| 2 | Coronary Ather?oscleros?s.mp. | 17,868 |
| 3 | Secondary prevention/ and (cardiovascular diseases/ or myocardial infarction.mp. or myocardial ischemia.mp.) | 5,214 |
| 4 | ((previous or prior or history of or following or post) adj (myocardial infarction or myocardial ischemia)).mp. | 25,777 |
| 5 | ((previous or prior or history of or after or following or post) adj (PCI or percutaneous coronary intervention or CABG or coronary artery bypass graft*)).mp. | 40,964 |
| 6 | 1 or 2 or3 or 4 or 5 | 439,014 |
| 7 | antithrombins/ | 14,742 |
| 8 | noac$.mp. | 4,752 |
| 9 | new oral anticoagulant$.mp. | 4,252 |
| 10 | doac$.mp. | 2,302 |
| 11 | direct oral anticoagulant$.mp. | 3,254 |
| 12 | New orally active anticoagulant$.mp. | 21 |
| 13 | Novel oral anticoagulant$.mp. | 2,929 |
| 14 | IIa inhibitor$.mp. | 287 |
| 15 | direct thrombin inhibitor$.mp. | 5,833 |
| 16 | Factor Xa Inhibitor/ | 7,247 |
| 17 | Xa inhibitor$.mp. | 8,151 |
| 18 | fxa inhibitor$.mp. | 1,150 |
| 19 | factor 10a inhibitor$.mp. | 3,711 |
| 20 | Dabigatran/ | 12,354 |
| 21 | (dabigatran or BIBR$1048 or pradax* or prazax*).mp. | 17,078 |
| 22 | (edoxaban or DU$176b or lixiana).mp. | 3,703 |
| 23 | (apixaban or BMS$562247 or eliquis).mp. | 48,049 |
| 24 | Rivaroxaban/ | 13,566 |
| 25 | (rivaroxaban or BAY 59$7939 or xarelto).mp. | 15,942 |
| 26 | (betrixaban or PRT054021).mp. | 486 |
| 27 | Or/7-26 | 89,683 |
| 28 | Platelet aggregation inhibitors/ | 68,441 |
| 29 | (antiplatelet* or anti-platelet* or thienopyridine or platelet inhibition).mp. | 78,136 |
| 30 | (clopidogrel or plavix).mp. | 66,472 |
| 31 | Prasugrel/ | 7,884 |
| 32 | (prasugrel or effient).mp. | 9,043 |
| 33 | (ticlopidine or ticlid).mp. | 25,030 |
| 34 | Dipyridamole/ | 31,030 |
| 35 | (dipyridamole or persantine).mp. | 35,556 |
| 36 | Aspirin/ or (aspirin or acetylsalicyl* or acetyl-salicyl* or acetyl salicyl*).mp. | 272,361 |
| 37 | (Ticagrelor or brilinta).mp. | 8,078 |
| 38 | (Triflusal or disgren).mp. | 806 |
| 39 | Or/28-38 | 380,934 |
| 40 | Heparin/ | 192,134 |
| 41 | Heparin.mp. | 279,182 |
| 42 | Heparin, Low-Molecular-Weight/ | 42,570 |
| 43 | (LMWH or low molecular weight heparin or nadroparin or fraxiparin or enoxaparin or clexane or lovenox or dalteparin or fragmin or ardeparin or normiflo or tinzaparin or logiparin or innohep or certoparin or sandoparin or reviparin or clivarin or danaparoid or orgaran).mp. | 71,224 |
| 44 | 40 or 41 or 42 or 43 | 293,744 |
| 45 | Coumarins/ | 26,215 |
| 46 | Warfarin/ | 100,880 |
| 47 | (warfarin or coumadin or acenocumarol or phenprocumon or 4-hydroxicoumarins or oral anticoagulant or vitamin K antagonist or VKA).mp. | 125,559 |
| 48 | 45 or 46 or 47 | 150,579 |
| 49 | (vorapaxar or zontivity).mp. | 977 |
| 50 | (cilostazol or pletal).mp. | 6,806 |
| 51 | (fondaparinux or arixtra).mp. | 8,727 |
| 52 | 49or 50 or 51 | 16,292 |
| 53 | 27 or 39 or 44 or 48 or 52 | 771,500 |
| 54 | Randomised controlled trials as Topic/ | 196,661 |
| 55 | Randomised controlled trial/ | 957,931 |
| 56 | Random allocation/ | 169,834 |
| 57 | Double blind method/ | 272,292 |
| 58 | Single blind method/ | 54,592 |
| 59 | clinical trial/ | 1,498,533 |
| 60 | clinical trial, phase i.pt. | 18,096 |
| 61 | clinical trial, phase ii.pt. | 29,216 |
| 62 | clinical trial, phase iii.pt. | 13,655 |
| 63 | clinical trial, phase iv.pt. | 1,493 |
| 64 | controlled clinical trial.pt. | 93,575 |
| 65 | randomised controlled trial.pt. | 461,456 |
| 66 | multicenter study.pt. | 232,720 |
| 67 | clinical trial.pt. | 516,361 |
| 68 | exp Clinical Trials as topic/ | 578,252 |
| 69 | (clinical adj trial$).tw. | 733,739 |
| 70 | ((singl$ or doubl$ or treb$ or tripl$) adj (blind$3 or mask$3)).tw. | 370,332 |
| 71 | PLACEBOS/ | 302,134 |
| 72 | placebo$.tw. | 468,815 |
| 73 | randomly allocated.tw. | 53,737 |
| 74 | Randomization/ | 173,076 |
| 75 | Single blind procedure/ | 30,956 |
| 76 | Double blind procedure/ | 148,980 |
| 77 | Crossover procedure/ | 55,269 |
| 78 | Placebo/ | 324,975 |
| 79 | Randomi?ed controlled trial$.tw. | 314,072 |
| 80 | Rct.tw. | 43,562 |
| 81 | Random allocation.tw. | 3,267 |
| 82 | Allocated randomly.tw. | 4,415 |
| 83 | (allocated adj2 random$).tw. | 60,378 |
| 84 | Single blind$.tw. | 36,616 |
| 85 | Double blind$.tw. | 328,121 |
| 86 | ((treble or triple) adj blind$).tw. | 1,413 |
| 87 | Prospective study/ | 904,037 |
| 88 | Or/54-87 | 4,049,886 |
| 89 | case report.tw. | 632,160 |
| 90 | Case study/ | 1,949,125 |
| 91 | letter/ | 1,960,867 |
| 92 | historical article/ | 348,690 |
| 93 | Abstract report/ | 91,079 |
| 94 | 89 or 90 or 91 or 92 or 93 | 4,564,258 |
| 95 | 88 not 94 | 3,936,337 |
| 96 | 6 and 53 and 95 | 19,261 |
| 97 | Limit 96 to humans | 18,068 |
| 98 | Remove duplicates from 97 | 15,134 |

Search was run on 01/03/2018 with Embase 1974 to 2018 February 28, Database Info Icon Ovid MEDLINE(R) Epub Ahead of Print, In-Process & Other Non-Indexed Citations, Ovid MEDLINE(R) Daily, Ovid MEDLINE and Versions(R)

**Table S2 Search strategy – CENTRAL (March 2018)**

| ID | Search terms | Hits |
| --- | --- | --- |
| #1 | MeSH descriptor: [Coronary Artery Disease] explode all trees | 4,102 |
| #2 | coronary artery disease | 16,762 |
| #3 | MeSH descriptor: [Peripheral Arterial Disease] explode all trees | 415 |
| #4 | Peripheral Artery Disease | 3,232 |
| #5 | Peripheral arterial Disease | 3,366 |
| #6 | Coronary Atherosclerosis | 2,843 |
| #7 | MeSH descriptor: [Secondary Prevention] explode all trees | 2,909 |
| #8 | MeSH descriptor: [Cardiovascular Diseases] explode all trees | 90,469 |
| #9 | myocardial infarction | 24,973 |
| #10 | myocardial ischemia | 5,991 |
| #11 | #7 and (#8 or #9 or #10) | 985 |
| #12 | (previous or prior or history or following or post) next (myocardial and (infarction or ischemia)) | 41 |
| #13 | (previous or prior or history of or after or following or post) next (PCI or percutaneous coronary intervention or CABG or coronary artery bypass graft) | 2,588 |
| #14 | #1 or #2 or #3 or #4 or #5 or #6 or #11 or #12 or #13 | 23,475 |
| #15 | MeSH descriptor: [Antithrombins] explode all trees | 726 |
| #16 | noac | 147 |
| #17 | new oral anticoagulant | 670 |
| #18 | doac | 76 |
| #19 | direct oral anticoagulant | 502 |
| #20 | New orally active anticoagulant | 125 |
| #21 | Novel oral anticoagulant | 177 |
| #22 | IIa inhibitor | 290 |
| #23 | direct thrombin inhibitor | 412 |
| #24 | Xa inhibitor | 576 |
| #25 | MeSH descriptor: [Factor Xa Inhibitors] explode all trees | 392 |
| #26 | fxa inhibitor | 74 |
| #27 | factor 10a inhibitor | 210 |
| #28 | dabigatran or BIBR 1048 or pradax or prazax | 742 |
| #29 | MeSH descriptor: [Dabigatran] explode all trees | 132 |
| #30 | edoxaban or DU 176b or lixiana | 337 |
| #31 | apixaban or BMS 562247 or eliquis | 577 |
| #32 | rivaroxaban or BAY 59 7939 or xarelto | 934 |
| #33 | MeSH descriptor: [Rivaroxaban] explode all trees | 197 |
| #34 | betrixaban or PRT054021 | 79 |
| #35 | or/12-34 | 3,518 |
| #36 | antiplatelet or anti-platelet or thienopyridine or platelet inhibition | 6,250 |
| #37 | MeSH descriptor: [Platelet Aggregation Inhibitors] explode all trees | 3,835 |
| #38 | clopidogrel or plavix | 4,106 |
| #39 | prasugrel or effient | 747 |
| #40 | ticlopidine or ticlid | 2,295 |
| #41 | MeSH descriptor: [Prasugrel Hydrochloride] explode all trees | 226 |
| #42 | dipyridamole or persantine | 1,373 |
| #43 | MeSH descriptor: [Dipyridamole] explode all trees | 604 |
| #44 | MeSH descriptor: [Aspirin] explode all trees | 5,048 |
| #45 | aspirin or acetylsalicyl or acetyl-salicyl or acetyl salicyl | 11,120 |
| #46 | Ticagrelor or brilinta | 762 |
| #47 | Triflusal or disgren | 135 |
| #48 | or/28-47 | 18,201 |
| #49 | MeSH descriptor: [Heparin] explode all trees | 4,519 |
| #50 | heparin | 10,420 |
| #51 | LMWH or low molecular weight heparin or nadroparin or fraxiparin or enoxaparin or clexane or lovenox or dalteparin or fragmin or ardeparin or normiflo or tinzaparin or logiparin or innohep or certoparin or sandoparin or reviparin or clivarin or danaparoid or orgaran | 5,094 |
| #52 | Heparin Low-Molecular-Weight | 3,439 |
| #53 | or/49-52 | 11,596 |
| #54 | MeSH descriptor: [Coumarins] explode all trees | 2,028 |
| #55 | MeSH descriptor: [Warfarin] explode all trees | 1,504 |
| #56 | warfarin or coumadin or acenocumarol or phenprocumon or 4-hydroxicoumarins or oral anticoagulant or vitamin K antagonist or VKA | 5,751 |
| #57 | or/54-56 | 6,188 |
| #58 | vorapaxar or zontivity | 118 |
| #59 | cilostazol or pletal | 629 |
| #60 | fondaparinux or arixtra | 421 |
| #61 | #58 or #59 or #60 | 1,143 |
| #62 | #35 or #48 or #53 or #57 or #61 | 32,063 |
| #63 | #62 and #14 | 4,537 |
| #64 | #63 in Trials | 3,691 |

Search was run 01/03/2018. All text and words variations were searched.

**Table S3 Study inclusion criteria (N=4)**

| No. | Study | Inclusion criteria |
| --- | --- | --- |
| 1 | CAPRIE [1] | Ischaemic stroke (including retinal and lacunar infarction):   - Focal neurological deficit likely to be of atherothrombotic origin - Onset ≥ 1 week and ≤ 6 months before randomisation - Neurological signs persisting 1 week from stroke onset CT or MRI ruling out haemorrhage or non-relevant disease   Myocardial infarction:   - Onset ≤ 35 days before randomisation - Two of: characteristic ischaemic pain for ≥ 20 min Elevation of CK, CK-MB, LDH, or AST to 2x upper limit of laboratory normal with no other explanation, development of new ≥40 Q waves in at least two adjacent ECG leads or new dominant R wave in V1 (R≥1 mm > S in V1)   Atherosclerotic peripheral arterial disease:  Intermittent claudication (WHO: leg pain on walking, arterial disease disappearing in <10 min on standing) of presumed atherosclerotic origin; and ankle/arm systolic BP ratio ≤0.85 in either leg at rest (two assessments on separate days);  Or history of intermittent claudication with previous leg amputation, reconstructive surgery, or angioplasty with no persisting complications from intervention |
| 2 | CHARISMA, NCT00050817 [2] | Patients were eligible to enrol in the trial if they were 45 years of age or older and had one of the following conditions:  Multiple atherothrombotic risk factors (to meet the criterion for enrolment on the basis of multiple risk factors, patients were required to have two major or three minor or one major and two minor atherothrombotic risk factors):   - - Major risk factors:     - Type 1 or 2 diabetes (with drug therapy)     - Diabetic nephropathy     - Ankle–brachial index <0.9     - Asymptomatic carotid stenosis ≥70% of luminal diameter     - ≥1 carotid plaque, as evidenced by intima–media thickness   - Minor risk factors:     - Systolic blood pressure ≥150 mm Hg, despite therapy for at least 3 months     - Primary hypercholesterolemia     - Current smoking >15 cigarettes/day     - Male sex and age ≥65 yr or female sex and age ≥70 yr   Documented CAD   - - Angina with documented multivessel coronary disease   - History of multivessel PCI   - History of multivessel CABG   - Myocardial infarction   Documented cerebrovascular disease   - - TIA during previous 5 yr   - IS during previous 5 yr   Documented symptomatic PAD   - - Current intermittent claudication and ankle–brachial index ≤0.85   History of intermittent claudication and previous intervention (e.g., amputation, peripheral bypass, or angioplasty) |
| 3 | COMPASS, NCT01776424 [3] | Patients were eligible for inclusion if they had CAD (CAD was defined as MI within the last 20 years, or multi-vessel coronary disease with symptoms or with history of stable or unstable angina, or multi-vessel PCI, or multi-vessel CABG surgery or PAD (PAD was defined as previous aorto-femoral bypass surgery, limb bypass surgery, or percutaneous transluminal angioplasty revascularization of the iliac, or infra-inguinal arteries, or previous limb or foot amputation for arterial vascular disease, or history of intermittent claudication or previous carotid revascularization or symptomatic carotid artery stenosis) and meet at least one of the following criteria:  Age ≥65 years  Age <65 years with disease in two vascular beds or at least 2 additional cardiovascular risk factors  Additional cardiovascular risk factors were:  Current smoker  Diabetes mellitus  Renal dysfunction with estimated glomerular filtration rate <60 ml/min  HF  Non-lacunar ischemic stroke ≥1 month ago |
| 4 | TRA 2°P–TIMI 50, NCT00526474 [4] | Eligible patients was at least 18 years and had history of:  Atherosclerosis, which was defined as a spontaneous MI  Or IS within the previous 2 weeks to 12 months  Or PAD associated with a history of intermittent claudication in conjunction with either an ankle–brachial index of less than 0.85  Or previous revascularization for limb ischemia. |

ABI, ankle branchial index; AST, aspartate transaminase; BP, blood pressure; CABG, coronary artery bypass graft; CAD, coronary artery disease; CK, creatine kinase; CK-MB, creatine kinase-MB; CV, cardiovascular; CT, Computed tomography; ECG, electrocardiography; HF, heart failure; IMT, intima media thickness; IS, ischemic stroke; LDH, Lactate dehydrogenase; MI, myocardial infarction; MRI, Magnetic resonance imaging; PCI, percutaneous coronary intervention; SBP, systolic blood pressure; TIA, transient ischemic attack; PAD, peripheral artery disease; WHO, World Health Organization; yr, years.

**Table S4 Risk of bias assessment (N=4)**

| Trial number (acronym) | | CAPRIE [1] | CHARISMA, NCT00050817 [2] | COMPASS, NCT01776424, [3] | TRA 2°P–TIMI 50, NCT00526474, [4] |
| --- | --- | --- | --- | --- | --- |
| Bias arising from the randomisation process | Was the allocation sequence random? | Yes | Yes | Yes | Yes |
|  | Was the allocation sequence concealed until participants were enrolled and assigned to interventions? | Yes | Yes | Yes | Yes |
|  | Did baseline differences between intervention groups suggest a problem with the randomisation process? | No | No | No | No |
|  | Risk-of-bias judgement | Low risk | Low risk | Low risk | Low risk |
| Bias due to deviations from intended interventions | Whether participants, carers and people delivering the interventions were blinded | Yes | Yes | Yes | Yes |
|  | Were carers and people delivering the interventions aware of participants' assigned intervention during the trial? | No | No | No | No |
|  | Were there deviations from the intended intervention that arose because of the experimental context? | No | No | No | No |
|  | Were these deviations from intended intervention balanced between groups? | NA | NA | NA | NA |
|  | Were these deviations likely to have affected the outcome? | NA | NA | NA | NA |
|  | Was an appropriate analysis used to estimate the effect of assignment to intervention? | Yes | Yes | NA | NA |
|  | Was there potential for a substantial impact (on the result) of the failure to analyse participants in the group to which they were randomised? | NA | NA | NA | NA |
|  | Risk-of-bias judgement | Low risk | Low risk | Low risk | Low risk |
| Bias due to missing outcome data | Were data for this outcome available for all, or nearly all, participants randomised? | Yes | Yes | Yes | Yes |
|  | Is there evidence that result was not biased by missing outcome data? | NA | NA | NA | NA |
|  | Could missingness in the outcome depend on its true value? | NA | NA | NA | NA |
|  | Do the proportions of missing outcome data differ between intervention groups? | NA | NA | NA | NA |
|  | Is it likely that missingness in the outcome depended on its true value? | NA | NA | NA | NA |
|  | Risk-of-bias judgement | Low risk | Low risk | Low risk | Low risk |
| Bias in measurement of the outcome | Was the method of measuring the outcome inappropriate? | No | No | No | No |
|  | Could measurement or ascertainment of the outcome have differed between intervention groups? | No | No | No | No |
|  | Were outcome assessors aware of the intervention received by study participants? | No | No | No | No |
|  | Could assessment of the outcome have been influenced by knowledge of intervention received? | NA | NA | NA | NA |
|  | Is it likely that assessment of the outcome was influenced by knowledge of intervention received? | NA | NA | NA | NA |
|  | Risk-of-bias judgement | Low risk | Low risk | Low risk | Low risk |
| Bias in selection of the reported result | Was the trial analysed in accordance with a pre-specified plan that was finalized before unblinded outcome data were available for analysis? | Yes | Yes | Yes | Yes |
|  | Is the numerical result being assessed likely to have been selected, on the basis of the results, from multiple outcome measurements (e.g. scales, definitions, time points) within the outcome domain? | No | No | No | No |
|  | Is the numerical result being assessed likely to have been selected, on the basis of the results, from multiple analyses of the data? | No | No | No | No |
|  | **Risk-of-bias judgements** | Low risk | Low risk | Low risk | Low risk |

NA – Not applicable

**Table S5 Other efficacy and safety results**

| Outcome | Study | Follow-up (months) | Interventions | N | N with event | % with event | Comparison  HR (LCI;UCI) |
| --- | --- | --- | --- | --- | --- | --- | --- |
| CHD death | COMPASS, NCT01776424 [3] | Mean: 23 | Rivaroxaban 5 mg BID | 9117 | 128 | 1.40 | 1.09 (0.85; 1.41) |
|  |  |  | Rivaroxaban 2.5 mg BID + ASA 100 mg od | 9152 | 86 | 0.90 | 0.73 (0.55; 0.96) |
|  |  |  | ASA 100 mg od | 9126 | 117 | 1.30 | Ref. |
| MI | CAPRIE [1] | Mean: 22.9 | Clopidogrel 75 mg od | 9553 | 275‡ | 2.90‡ | 0.82 (0.70; 0.96)‡ |
|  |  |  | ASA 325 mg od | 9546 | 333‡ | 3.50‡ | Ref. |
|  | COMPASS, NCT01776424 [3] | Mean: 23 | Rivaroxaban 5 mg BID | 9117 | 182 | 2.00 | 0.89 (0.73; 1.08) |
|  |  |  | Rivaroxaban 2.5 mg BID + ASA 100 mg od | 9152 | 178 | 1.90 | 0.86 (0.70; 1.05) |
|  |  |  | ASA 100 mg od | 9126 | 205 | 2.20 | Ref. |
|  | TRA 2°P–TIMI 50, NCT00526474 [4] | Median: 30 | Vorapaxar 2.5 mg od | 13225 | 564 | 5.20† | 0.83 (0.74; 0.93) |
|  |  |  | Placebo | 13224 | 673 | 6.10† | Ref. |
| Stroke | COMPASS, NCT01776424 [3] | Mean: 23 | Rivaroxaban 5 mg BID | 9117 | 117 | 1.30 | 0.82 (0.65; 1.05) |
|  |  |  | Rivaroxaban 2.5 mg BID + ASA 100 mg od | 9152 | 83 | 0.90 | 0.58 (0.44; 0.76) |
|  |  |  | ASA 100 mg od | 9126 | 142 | 1.60 | Ref. |
|  | TRA 2°P–TIMI 50, NCT00526474 [4] | Median: 30 | Vorapaxar 2.5 mg od | 13225 | 315 | 2.80† | 0.97 (0.83; 1.14) |
|  |  |  | Placebo | 13224 | 324 | 2.80† | Ref. |
| ALI | COMPASS, NCT01776424 [3] | Mean: 23 | Rivaroxaban 5 mg BID | 9117 | 24 | 0.30 | 0.60 (0.36; 1) |
|  |  |  | Rivaroxaban 2.5 mg BID + ASA 100 mg od | 9152 | 22 | 0.20 | 0.55 (0.32; 0.92) |
|  |  |  | ASA 100 mg od | 9126 | 40 | 0.40 | Ref. |
| Amputations | COMPASS, NCT01776424 [3] | Mean: 23 | Rivaroxaban 5 mg BID | 9117 | 30 | 0.30 | 0.64 (0.40; 1.01) |
|  |  |  | Rivaroxaban 2.5 mg BID + ASA 100 mg od | 9152 | 30 | 0.30 | 0.64 (0.40; 1.00) |
|  |  |  | ASA 100 mg od | 9126 | 47 | 0.50 | Ref. |
| HF | COMPASS, NCT01776424 [3] | Mean: 23 | Rivaroxaban 5 mg BID | 9117 | 191 | 2.10 | 0.99 (0.81; 1.21) |
|  |  |  | Rivaroxaban 2.5 mg BID + ASA 100 mg od | 9152 | 197 | 2.20 | 1.02 (0.84; 1.24) |
|  |  |  | ASA 100 mg od | 9126 | 192 | 2,10 | Ref. |
| Unstable angina | COMPASS, NCT01776424 [3] | Mean: 23 | Rivaroxaban 5 mg BID | 9117 | 28 | 0.30 | 0.90 (0.54; 1.50) |
|  |  |  | Rivaroxaban 2.5 mg BID + ASA 100 mg od | 9152 | 24 | 0.30 | 0.77 (0.45; 1.31) |
|  |  |  | ASA 100 mg od | 9126 | 31 | 0.30 | Ref. |
| Revascularization | COMPASS, NCT01776424 [3] | Mean: 23 | Rivaroxaban 5 mg BID | 9117 | 590 | 6.50 | 0.93 (0.83; 1.04) |
|  |  |  | Rivaroxaban 2.5 mg BID + ASA 100 mg od | 9152 | 595 | 6.50 | 0.93 (0.83; 1.04) |
|  |  |  | ASA 100 mg od | 9126 | 632 | 6.90 | Ref. |
|  | TRA 2°P–TIMI 50, NCT00526474 [4] | Median: 30 | Vorapaxar 2.5 mg od | 13225 | — | 13.60† | 0.89 (0.83; 0.95) |
|  |  |  | Placebo | 13224 | — | 15.50† | Ref. |
| Graft failure | COMPASS, NCT01776424 [3] | Mean: 23 | Rivaroxaban 5 mg bid | 483 | 61 | 12.60 | 0.97 (0.68; 1.39)‡ |
|  |  |  | Rivaroxaban 2.5 mg bid + ASA 100 mg od | 502 | 70 | 13.90 | 1.08 (0.77; 1.53)‡ |
|  |  |  | ASA 100 mg od | 463 | 60 | 13.00 | Ref. |
| Stent thrombosis | COMPASS, NCT01776424 [3] | Mean: 23 | Rivaroxaban 5 mg bid | 9117 | 52 | 0.60 | 1.11 (0.75; 1.64) |
|  |  |  | Rivaroxaban 2.5 mg bid + ASA 100 mg od | 9152 | 52 | 0.60 | 1.10 (0.74; 1.63) |
|  |  |  | ASA 100 mg od | 9126 | 47 | 0.50 | Ref. |
|  | TRA 2°P–TIMI 50, NCT00526474 [4] | Median: 30 | Vorapaxar 2.5 mg od | 7223 | 72 | 1.20† | 0.74 (0.55; 1.01) |
|  |  |  | Placebo | 7268 | 97 | 1.50† | Ref. |
| Fatal Bleeding | CAPRIE [1] | Mean: 22.9 | Clopidogrel 75 mg od | 9599 | 23 | 0.24‡ | 0.85 (0.49; 1.48)‡ |
|  |  |  | ASA 325 mg od | 9586 | 27 | 0.28‡ | Ref. |
|  | CHARISMA, NCT00050817 [2] | Median:28 | Clopidogrel 75 mg od + ASA 75-162 mg od | 7802 | 26 | 0.30 | 1.53 (0.83; 2.82) |
|  |  |  | ASA 75-162 mg od | 7801 | 17 | 0.20 | Ref. |
|  | COMPASS, NCT01776424 [3] | Mean: 23 | Rivaroxaban 5 mg bid | 9117 | 14 | 0.20 | 1.40 (0.62; 3.15) |
|  |  |  | Rivaroxaban 2.5 mg bid + ASA 100 mg od | 9152 | 15 | 0.20 | 1.49 (0.67; 3.33) |
|  |  |  | ASA 100 mg od | 9126 | 10 | 0.10 | Ref. |
|  | TRA 2°P–TIMI 50, NCT00526474 [4] | Median: 30 | Vorapaxar 2.5 mg od | 13186 | 29 | 0.30† | 1.46 (0.82; 2.58) |
|  |  |  | Placebo | 13166 | 20 | 0.20† | Ref. |
| Intracranial bleeding | CAPRIE [1] | Mean: 22.9 | Clopidogrel 75 mg od | 9599 | 34 | 0.35 | 0.72 (0.46; 1.12)‡ |
|  |  |  | ASA 325 mg od | 9586 | 47 | 0.49 | Ref. |
|  | CHARISMA, NCT00050817 [2] | Median:28 | Clopidogrel 75 mg od + ASA 75-162 mg od | 7802 | 26 | 0.30 | 0.96 (0.56; 1.65) |
|  |  |  | ASA 75-162 mg od | 7801 | 27 | 0.30 | Ref. |
|  | COMPASS, NCT01776424 [3] | Mean: 23 | Rivaroxaban 5 mg bid | 9117 | 43‡ | 0.47‡ | 1.80 (1.09; 2.96)‡ |
|  |  |  | Rivaroxaban 2.5 mg bid + ASA 100 mg od | 9152 | 31‡ | 0.34‡ | 1.16 (0.67; 2.00)‡ |
|  |  |  | ASA 100 mg od | 9126 | 28‡ | 0.31‡ | Ref. |
|  | TRA 2°P–TIMI 50, NCT00526474 [4] | Median: 30 | Vorapaxar 2.5 mg od | 13186 | 102 | 1.00† | 1.94 (1.39; 2.70) |
|  |  |  | Placebo | 13166 | 53 | 0.50† | Ref. |
| Bleeding requiring re-operation | COMPASS, NCT01776424[3] | Mean: 23 | Rivaroxaban 5 mg bid | 9117 | 16 | 0.20 | 2.00 (0.86; 4.67) |
|  |  |  | Rivaroxaban 2.5 mg bid + ASA 100 mg od | 9152 | 10 | 0.10 | 1.24 (0.49; 3.14) |
|  |  |  | ASA 100 mg od | 9126 | 8 | <0.10 | Ref. |
| Bleeding requiring hospitalization | CAPRIE [1] | Mean: 22.9 | Clopidogrel 75 mg od | 9553 | 169 | 1.80 | 0.82 (0.67; 1.01)‡ |
|  |  |  | ASA 325 mg od | 9546 | 205 | 2.20 | Ref. |
|  | COMPASS, NCT01776424 [3] | Mean: 23 | Rivaroxaban 5 mg bid | 9117 | 154 | 1.70 | 1.42 (1.11; 1.81) |
|  |  |  | Rivaroxaban 2.5 mg bid + ASA 100 mg od | 9152 | 208 | 2.30 | 1.91 (1.51; 2.41) |
|  |  |  | ASA 100 mg od | 9126 | 109 | 1.20 | Ref. |
|  | TRA 2°P–TIMI 50, NCT00526474 [4] | Median: 30 | Vorapaxar 2.5 mg od | 13186 | 1392 | 12.50† | 1.44 (1.33; 1.56) |
|  |  |  | Placebo | 13166 | 992 | 8.90† | Ref. |
| Gastrointestinal bleeding | CAPRIE [1] | Mean: 22.9 | Clopidogrel 75 mg od | 9599 | 191 | 1.99 | 0.75 (0.62; 0.90)‡ |
|  |  |  | ASA 325 mg od | 9586 | 255 | 2.66 | Ref. |
|  | COMPASS, NCT01776424 [3] | Mean: 23 | Rivaroxaban 5 mg bid | 9117 | 338‡ | 3.71‡ | 1.40 (1.02; 1.95)‡ |
|  |  |  | Rivaroxaban 2.5 mg bid + ASA 100 mg od | 9152 | 393‡ | 4.29‡ | 2.15 (1.60; 2.89)‡ |
|  |  |  | ASA 100 mg od | 9126 | 211‡ | 2.31‡ | Ref. |
| Total SAEs | COMPASS, NCT01776424 [3] | Mean: 23 | Rivaroxaban 5 mg bid | 9117 | 773 | 8.50 | 1.08 (0.98; 1.20)‡ |
|  |  |  | Rivaroxaban 2.5 mg bid + ASA 100 mg od | 9152 | 786 | 8.60 | 1.10 (0.99; 1.22)‡ |
|  |  |  | ASA 100 mg od | 9126 | 716 | 7.80 | Ref. |
|  | TRA 2°P–TIMI 50, NCT00526474 [4] | Median: 30 | Vorapaxar 2.5 mg od | 13186 | 3514 | 26.65 | 1.03 (0.98; 1.08)‡ |
|  |  |  | Placebo | 13166 | 3419 | 25.97 | Ref. |
| Any discontinuation | CAPRIE [1] | Mean: 22.9 | Clopidogrel 75 mg od | 9599 | 2044‡ | 21.30 | 1.01 (0.95; 1.07)‡ |
|  |  |  | ASA 325 mg od | 9586 | 2023‡ | 21.10 | Ref. |
|  | CHARISMA, NCT00050817 [2] | Median:28 | Clopidogrel 75 mg od + ASA 75-162 mg od | 7802 | 26 | 0.30 | 0.96 (0.56; 1.65) |
|  |  |  | ASA 75-162 mg od | 7801 | 27 | 0.30 | Ref. |
|  | COMPASS, NCT01776424 [3] | Mean: 23 | Rivaroxaban 5 mg bid | 9117 | 1637 | 17.50 | 1.11 (1.03; 1.19)‡ |
|  |  |  | Rivaroxaban 2.5 mg bid + ASA 100 mg od | 9152 | 1604 | 16.90 | 1.08 (1.01; 1.16)‡ |
|  |  |  | ASA 100 mg od | 9126 | 1491 | 15.90 | Ref. |
|  | TRA 2°P–TIMI 50, NCT00526474 [4] | Median: 30 | Vorapaxar 2.5 mg od | 13186 | 5407 | 41.00‡ | 1.05 (1.01; 1.09)‡ |
|  |  |  | Placebo | 13166 | 5196 | 39.50‡ | Ref. |
| Discontinuations due to AEs | CAPRIE [1] | Mean: 22.9 | Clopidogrel 75 mg od | 9599 | 1146‡ | 11.94 | 1.00 (0.92; 1.09)‡ |
|  |  |  | ASA 325 mg od | 9586 | 1143‡ | 11.92 | Ref. |
|  | CHARISMA, NCT00050817 [2] | Median:28 | Clopidogrel 75 mg od + ASA 75-162 mg od | 7802 | 374 | 4.80 | 0.98 (0.85; 1.13)‡ |
|  |  |  | ASA 75-162 mg od | 7801 | 382 | 4.90 | Ref. |
|  | COMPASS, NCT01776424 [3] | Mean: 23 | Rivaroxaban 5 mg bid | 9117 | 307 | 3.40 | 1.20 (1.09; 1.54)‡ |
|  |  |  | Rivaroxaban 2.5 mg bid + ASA 100 mg od | 9152 | 312 | 3.40 | 1.31 (1.11; 1.55)‡ |
|  |  |  | ASA 100 mg od | 9126 | 238 | 2.60 | Ref. |

†Kaplan-Meier estimate, ‡Calculated on the basis of available data

ASA, acetylsalicylic acid; AE, adverse event, ALI, acute limb ischemia; bid, bis in die = twice a day; CHD, coronary heart disease; HF, heart failure; HR, hazard ratio; IS, Ischemic stroke; LCI, lower confidence interval; MI, myocardial infraction; od, once a day RRR, relative risk reduction; UCI, upper confidence interval.

**Table S6 List of included publications (N=27, 4 studies)**

| No. | Study, primary reference | Secondary references |
| --- | --- | --- |
| 1 | COMPASS [3], NCT01776424 | S. J. Connolly et al. 2018 [5]  S. S. Anand et al. 2018 [6] |
| 2 | CAPRIE [1] | D. L. Bhatt et al. 2000 [7]  L. A. Harker et al. 2012 [8]  P. A. Ringleb et al. 2004 [9] |
| 3 | CHARISMA [2], NCT00050817 | D. L. Bhatt et al. 2007 [10]  P. P. Cacoub et al. 2009 [11] |
| 4 | TRA 2°P–TIMI 50 [4], NCT00526474 | E. A. Bohula et al. 2015 [12]  B. M. Scirica et al. 2012 [13]  G. Magnani et al. 2017 [14]  D. A. Morrow et al. 2013 [15]  M. P. Bonaca et al. 2013 [16]  D. D. Berg et al. 2016 [17]  E. A. Bohula et al. 2016 [18]  M. P. Bonaca et al. 2016 [19]  M. P. Bonaca et al. 2014 [20]  M. P. Bonaca et al. 2014 [21]  E.C Kosova et al. 2017 [22]  M. P. Bonaca et al. 2016 [23]  S. K. Kidd et al. 2016 [24]  E. A. Bohula et al. 2015 [12]  M. A. Cavender et al. 2015 [25] |

**Supplementary references**

1. Caprie Steering Committee. "A randomised, blinded, trial of clopidogrel versus aspirin in patients at risk of ischaemic events (CAPRIE). CAPRIE Steering Committee," *Lancet*, vol. 348, no. 9038, pp. 1329–1339, 1996.

2. D. L. Bhatt, K. A. Fox, W. Hacke et al. "Clopidogrel and aspirin versus aspirin alone for the prevention of atherothrombotic events," *New England Journal of Medicine*, vol. 354, no. 16, pp. 1706–1717, 2006.

3. J. W. Eikelboom, S. J. Connolly, J. Bosch et al. "Rivaroxaban with or without aspirin in stable cardiovascular disease," *New England Journal of Medicine*, vol. 377, no. 14, pp. 1319–1330, 2017.

4. D. A. Morrow, E. Braunwald, M. P. Bonaca et al. "Vorapaxar in the secondary prevention of atherothrombotic events," *New England Journal of Medicine*, vol. 366, no. 15, pp. 1404–1413, 2012.

5. S. J. Connolly, J. W. Eikelboom, J. Bosch et al. "Rivaroxaban with or without aspirin in patients with stable coronary artery disease: an international, randomised, double-blind, placebo-controlled trial," *Lancet*, vol. 391, no. 10117, pp. 205–218, 2018.

6. S. S. Anand, J. Bosch, J. W. Eikelboom et al. "Rivaroxaban with or without aspirin in patients with stable peripheral or carotid artery disease: An international, randomised, double-blind, placebo-controlled trial," *Lancet*, vol. 391, no. 10117, pp. 219–229, 2018.

7. D. L. Bhatt, A. T. Hirsch, P. A. Ringleb, W. Hacke, E. J. Topol. "Reduction in the need for hospitalization for recurrent ischemic events and bleeding with clopidogrel instead of aspirin. CAPRIE investigators," *American Heart Journal*, vol. 140, no. 1, pp. 67–73, 2000.

8. L. A. Harker, J. P. Boissel, A. J. Pilgrim, M. Gent. "Comparative safety and tolerability of clopidogrel and aspirin: results from CAPRIE. CAPRIE Steering Committee and Investigators. Clopidogrel versus aspirin in patients at risk of ischaemic events," *Drug Safety*, vol. 21, no. 4, pp. 325–335, 1999.

9. P. A. Ringleb, D. L. Bhatt, A. T. Hirsch, E. J. Topol, W. Hacke, Clopidogrel Versus Aspirin in Patients at Risk of Ischemic Events Investigators. "Benefit of clopidogrel over aspirin is amplified in patients with a history of ischemic events," *Stroke*, vol. 35, no. 2, pp. 528–532, 2004.

10. D. L. Bhatt, M. D. Flather, W. Hacke et al. "Patients with prior myocardial infarction, stroke, or symptomatic peripheral arterial disease in the CHARISMA trial," *Journal of the American College of Cardiology*, vol. 49, no. 19, pp. 1982–1988, 2007.

11. P. P. Cacoub, D. L. Bhatt, P. G. Steg, E. J. Topol, M. A. Creager, C. Investigators. "Patients with peripheral arterial disease in the CHARISMA trial," *European Heart Journal*, vol. 30, no. 2, pp. 192–201, 2009.

12. E. A. Bohula, P. E. Aylward, M. P. Bonaca et al. "Efficacy and safety of vorapaxar with and without a thienopyridine for secondary prevention in patients with previous myocardial infarction and no history of stroke or transient ischemic attack: Results from TRA 2°P-TIMI 50," *Circulation*, vol. 132, no. 20, pp. 1871–1879, 2015.

13. B. M. Scirica, M. P. Bonaca, E. Braunwald et al. "Vorapaxar for secondary prevention of thrombotic events for patients with previous myocardial infarction: a prespecified subgroup analysis of the TRA 2°P-TIMI 50 trial," *Lancet*, vol. 380, no. 9850, pp. 1317–1324, 2012.

14. G. Magnani, M. P. Bonaca, E. Braunwald et al. "Efficacy and safety of vorapaxar as approved for clinical use in the United States," *J Am Heart Assoc*, vol. 4, no. 3, pp. e001505, 2017.

15. D. A. Morrow, M. J. Alberts, J. P. Mohr et al. "Efficacy and safety of vorapaxar in patients with prior ischemic stroke," *Stroke*, vol. 44, no. 3, pp. 6918, 2013.

16. M. P. Bonaca, B. M. Scirica, M. A. Creager et al. "Vorapaxar in patients with peripheral artery disease: results from TRA 2°P-TIMI 50," *Circulation*, vol. 127, no. 14, pp. 1522–1529, 1529e1521–1526, 2013.

17. D. D. Berg, M. P. Bonaca, E. Braunwald et al. "Outcomes in stable patients with previous atherothrombotic events receiving vorapaxar who experience a new acute coronary event (from TRA 2°P-TIMI 50)," *American Journal of Cardiology*, vol. 117, no. 7, pp. 1055–1058, 2016.

18. E. A. Bohula, M. P. Bonaca, E. Braunwald et al. "Atherothrombotic risk stratification and the efficacy and safety of vorapaxar in patients with stable ischemic heart disease and previous myocardial infarction," *Circulation*, vol. 134, no. 4, pp. 304–313, 2016.

19. M. P. Bonaca, M. A. Creager, J. Olin et al. "Peripheral revascularization in patients with peripheral artery disease with vorapaxar: Insights from the TRA 2°P-TIMI 50 trial," *JACC: Cardiovascular Interventions*, vol. 9, no. 20, pp. 2157–2164, 2016.

20. M. P. Bonaca, B. M. Scirica, E. Braunwald et al. "New ischemic stroke and outcomes with vorapaxar versus placebo: Results from the TRA 2°P-TIMI 50 trial," *Journal of the American College of Cardiology*, vol. 64, no. 22, pp. 2318–2326, 2014.

21. M. P. Bonaca, B. M. Scirica, E. Braunwald et al. "Coronary stent thrombosis with vorapaxar versus placebo: Results from the TRA 2°P-TIMI 50 trial," *Journal of the American College of Cardiology*, vol. 64, no. 22, pp. 2309–2317, 2014.

22. E. C. Kosova, M. P. Bonaca, M. Dellborg et al. "Vorapaxar in patients with coronary artery bypass grafting: findings from the TRA 2°P-TIMI 50 trial," *European Heart Journal: Acute Cardiovascular Care*, vol. 6, no. 2, pp. 164–172, 2017.

23. M. P. Bonaca, J. A. Gutierrez, M. A. Creager et al. "Acute limb ischemia and outcomes with vorapaxar in patients with peripheral artery disease," *Circulation*, vol. 133, no. 10, pp. 997–1005, 2016.

24. S. K. Kidd, M. Bonaca, B. Scirica et al. "Efficacy and safety of vorapaxar for secondary prevention in older patients: Observations from TRA 2°P-TIMI 50," *Journal of the American College of Cardiology*, vol. 67, no. 13 SUPPL. 1, pp. 2098, 2016.

25. M. A. Cavender, B. M. Scirica, M. P. Bonaca et al. "Vorapaxar in patients with diabetes mellitus and previous myocardial infarction: findings from the thrombin receptor antagonist in secondary prevention of atherothrombotic ischemic events-TIMI 50 trial," *Circulation*, vol. 131, no. 12, pp. 1047053, 2015.
